# A review on effect of nanoparticle addition on thermal behavior of natural fiber–reinforced composites

**DOI:** 10.1016/j.heliyon.2024.e41192

**Published:** 2024-12-13

**Authors:** D. Balaji, P. Sathish Kumar, V. Bhuvaneshwari, L. Rajeshkumar, Manoj Kumar Singh, M.R. Sanjay, Suchart Siengchin

**Affiliations:** a Department of Mechanical Engineering, KPR Institute of Engineering and Technology, Coimbatore, 641407, Tamil Nadu, India; b Centre for Research and Development, KPR Institute of Engineering and Technology, Coimbatore, 641407, Tamil Nadu, India; c Natural Composites Research Group Lab, Department of Materials and Production Engineering, The Sirindhorn International Thai-German Graduate School of Engineering, King Mongkut's University of Technology North Bangkok, Bangkok, 10800, Thailand; d AU-Sophisticated Testing and Instrumentation Centre and Department of Mechanical Engineering, Alliance School of Applied Engineering, Alliance University, Bengaluru, 562106, Karnataka, India

**Keywords:** Nanoparticle, Thermal behavior, Natural fibers, Polymer composites

## Abstract

Always, the environment in which humans live needs to be saved from various calamities, and one such calamity is usage of petroleum-based products. Petroleum-based products are derived from various synthetic processes that adversely affect the environment. It may not reflect immediately, but it affects in the near future. They are non-environmentally friendly and cannot progress toward the sustainability factor. The alternative to metallic or synthetic fibers is natural fibers that are derived from plant sources. The demerits of using natural fiber is its less strength; however, this strength can be enhanced by incorporating it as a strengthening component in polymer matrix composite (PMC) materials. Still, the major advantage of using metal is its ability to withstand higher temperatures, whereas PMCs fail in these characteristics. The use of nanoparticles as fillers in the natural fiber–reinforced PMCs is a probable solution to the above problem. This review assesses the thermal characteristics of various nanoparticle-filled natural fiber–based polymer composites. It can be seen from most of the research that the filled polymer composites exhibit better thermal behavior compared with non-filled polymer composites. This consolidation would be useful for researchers to further accelerate their research in this domain.

## Highlights

• This review highlights the role of nanoparticles in enhancing the thermal properties of natural fiber-based polymer composites.
• Previous studies have analyzed for improving thermal behavior of natural fiber-reinforced composites.
• Common properties tailoring techniques are organic and inorganic fillers addition, orientation adjustment, chemical modification.
• Thermal analysis of the composite materials paves the way for the development of advanced and novel composite materials.
• Nanoparticle-incorporated natural fiber composites exhibit better thermal behavior, making them ideal for high-temperature applications.

## Graphical abstract

**Figure:**
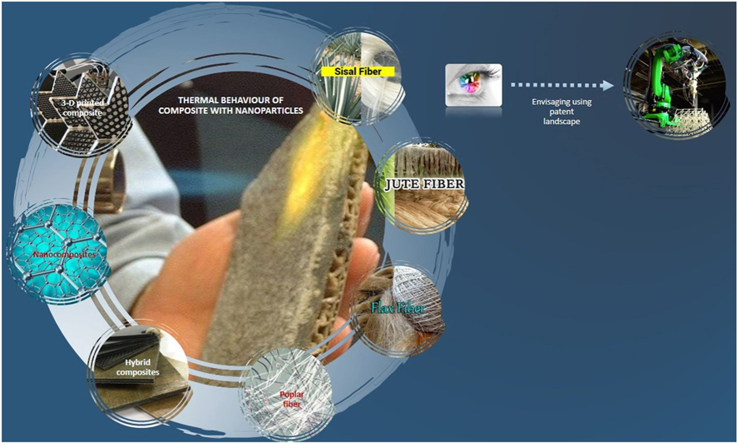

## 1 Introduction

An increased focus has been placed on the production of polymer composites reinforced with natural fibres over the past few decades. This is primarily due to concerns surrounding sustainability and degradability, as evidenced by various scholarly sources [1]. The use of natural fibers like Fique fibers are being proposed for creating composites that are lightweight and less expensive than traditional synthetic fibers, including carbon fiber, Kevlar, and fiber glass [[2], [3], [4], [5]]. Furthermore, reinforced polymer composites made from natural fibers may be an eco-friendly option. The mechanical and physical characteristics of pure cellulose fibers are influenced by their source and characteristics, including but not limited to diameter, length, and specific gravity. These properties play a pivotal role in identifying the potential applications of these fibers. The use of natural fibers, including sugar palm fiber, OPEFB fiber, kenaf fiber, flax fiber, pineapple leaf fiber, banana fiber, and jute in polymer matrix composites (PMCs) present numerous benefits, such as recyclability, reduced density, biodegradability, accessibility, enhanced mechanical and physical characteristics, and favorable thermal steadiness of the resultant materials [[6], [7], [8], [9]]. The use of natural fibers, including but not limited to kenaf, jute, and hemp, as reinforcement for polymer composites has resulted in exceptional thermal properties that are comparable to those of synthetic fibers. This has led to an expansion of their potential applications in various fields, including aerospace and construction industries, as evidenced by studies [[10], [11], [12], [13], [14]]. Before their implementation in structural applications, it is necessary to use testing methodologies to examine the composite structure and assess its performance under cyclic loading and thermal conditions.

The categorization of natural fiber can be delineated into two primary classifications based on its source: inorganic and organic. Typically, inorganic natural fibers consist of mineral fibers, including wollastonite, fibrous brucite, and asbestos. Various plant species, including grass, bamboo, wheat, and bagasse, can produce fibers from their stalks. The constituents of plant fibers primarily comprise three fundamental elements, namely cellulose, hemicelluloses, and lignin. The fibers in question are commonly known as lignocellulosic materials, because cellulose constitutes the primary chemical, alongside varying quantities of lignin and hemicellulose [15,16]. Mechanical characteristics of fibers are attributed to the hydrogen bonds present between macromolecules, as well as the orientation of crystalline cellulose fibrils within the cell wall. Lignin exhibits strong adherence to hemicellulose and forms cross-links with polysaccharides, thereby occupying the interstitial spaces within the cell wall matrix composed of pectin, cellulose, and hemicellulose constituents, thereby conferring mechanical stability to the cell wall [17,18].

The primary constraints associated with natural fibers pertain to their hydrophilic characteristics, which can augment moisture absorption and undermine the bonding interplay between the fibers and polymer matrices [[19], [20], [21], [22]]. The drawbacks associated with natural fibers, such as inadequate interfacial bonding and the manifestation of hydrophilic properties, can be overcome by using diverse chemical processing that modify the compatibility of the natural fibers with the matrix. The application of chemical processing results in the formation of natural fibers that are fully cured and cross-linked. These fibers serve to reinforce the polymeric matrix, thereby leading to enhanced mechanical strength and thermal stability. This has been documented in previous studies [23,24]. Improved compatibility among the natural fibers and polymer binder might be achieved through the efficient removal of surface contaminations and hydroxyl group constituents [25]. This phenomenon facilitates a robust chemical adhesion at the interfaces of two phases, resulting in a well-established network. This process has the potential to enhance the utility of natural fibers in various domains, including marine and automobile sectors, by augmenting their resistance to wear, tear, and creep [[26], [27], [28], [29]]. The augmentation of these characteristics has the potential to enhance the longevity of the material, particularly when subjected to severe circumstances, such as elevated temperatures, higher levels of humidity, and acidic environment [[30], [31], [32], [33]].

The investigation of thermal behavior under diverse conditions is a crucial aspect to be considered for the design and development of hybrid composites for multiple applications. The nonlinearity of thermal property fluctuations in hybrid composites presents a challenge in determining and forecasting thermal conductivity [[34], [35], [36]]. The incorporation of filler particles of silicon carbide into the pristine epoxy polymer composites results in an enhancement of thermal conductivity, which is directly proportional to the percentage of volume fraction. The thermal conductivity of silicon carbide in isolation is notably higher than that of hybrid composites. However, when combined with epoxy resin, silicon carbide exhibits reduced thermal conductivity due to the relatively diminutive size of the filler particles, limiting their efficacy in producing conductive pathways is limited [37]. Consequently, insufficient cooling may lead to the malfunction of electronic devices. The operational temperature range limit is a critical factor that determines the resilience and recital of electronic devices. Precise management of thermal conditions is a crucial factor in determining the high performance of semiconductors [38]. The efficacy of using high-performance computing systems is depends on the selection of filler materials with superior thermal conductivity. The use of hybrid nano-fillers in the modification of polymer matrices has opened up extensive avenues for research, enabling the attainment of superior thermal and mechanical properties applicable across various fields.

According to previous research, various nanoparticle fillers are frequently used to enhance the thermal conductivity and electrical resistivity of polymers [39]. Owing to the inherently low thermal conductivity of polymers, various fillers including mica, zinc oxide, glass fiber, boron nitride, aluminum nitride, silicon carbides, and alumina are incorporated [40]. The enhancement of thermal resistance can be observed in silica hybrid composites, which can be attributed to the robust interfacial bonding between silica particles and epoxy resin, as reported in Ref. [41]. The incorporation of graphene nanoplates (GNPs) at a concentration of 1 % by weight resulted in a notable improvement in thermal conductivity. This effect can be attributed to the uniform distribution of GNPs within the epoxy polymer matrix as well as the establishment of internal bonding. Some studies involved a comparative analysis of the experimental thermal conductivity values obtained for polymer composites filled with particulate matter, with the mathematical methods, namely the rule of mixture thermal conductivity model, parallel thermal conductivity model, and Maxwell thermal conductivity model [42,43]. To enhance the thermal conductivity and thermal stability of polymer composites, fillers including graphene, CNT, copper mesh and powder, can be incorporated into carbon fiber–reinforced polymer (CFRP) to create hybrid polymer composites [44].

The use of hybrid fillers including boron nitride, SiC, and aluminum nitride in polymer composites has been observed to be a more efficacious approach in reducing the CTE [45,46]. A few experiments were conducted by adding SiC nanoparticles into glass fiber-based epoxy composites to evaluate their thermal stability. The results of thermal experiments indicated that composites with 5 % and 20 % SiC contents exhibited relatively high thermal conductivities [47]. Thermogravimetric analysis (TGA) was conducted on composites comprising polyester resin, glass fiber, and jute fiber. The results indicated that the composite demonstrated a lower weight loss with increasing temperature [48]. In a TGA comparison study of hybrid polymer composites, carbon fiber composite, and glass fiber composite, it was found that the thermal stability of the hybrid composites was superior [49]. The TGA of the composite material consisting of PMMA toughened glass and epoxy revealed a greater degree of mass loss, whereas the incorporation of SiC helped to maintain its temperature stability [50]. The findings suggest that the incorporation of GNPs, graphene oxide (GO), reduced graphene oxide (rGO), and multi-walled carbon nanotubes (MWCNTs) results in an increase in thermal conductivity [51]. The impact of filler materials on the glass transition temperature of polymers is significant. Certain composites undergo testing for structural stability at low temperatures, as indicated in Ref. [52]. Hence, from all the above discussions it could be understood that that the incorporation of natural or inorganic nanoparticles exhibited better thermal characteristics when compared with the composites without nanoparticles. Accordingly, the focus of the current review article was to articulate the significance of nanoparticle addition to the natural fiber–based polymer composites in improving the thermal characteristics.

## 2 Thermal behavior of natural fiber-based polymer composites

### 2.1 Sisal fiber composites

Natural fiber polymer composites could be analyzed for their disintegration by temperature rise and weight loss in relation to temperature, including residue material beyond maximum heating, using TGA. The lignocellulosic fiber combined polymer matrix that makes up natural fiber composites. The rate at which these materials degrade in heat depends on the reinforcement and matrix used, with each component degrading at distinct temperatures. When something degrades at a certain temperature, it loses mass in accordance with that temperature. The process of reducing weight in fiber composites can be broken down into three distinct phases: first, because of the evaporation of water; second, owing to the breakdown of the fiber reinforcement; and third, because of de-polymerization in the binder itself. Recently, the hybridization process has enhanced the thermal resistance of fiber composites. Among the hybridization influences in the thermal resistance of sisal composites are the following. Researchers evaluated the thermal properties exhibited by the jute or sisal hybrid composites. As a result of the initial drying process, the composites lost approximately 5 wt%. The deterioration of binder and fiber caused 75 % of the composite's mass loss when the temperature began to rise. The composites completely degraded when subjected to high temperatures. Maximum thermal stability was observed in a composite made up of 50 % jute and 50 % sisal fiber. Additionally, alkaline treatment was found to be advantageous in improving the composites' thermal stability [53].

Gupta [54] obtained comparable findings from a different study that investigated identical jute or sisal epoxy composites with varying fiber weight fractions [54]. Similarly, the temperature resistance of sisal material was improved by glass fiber incorporation. This increase can be attributed in large part to the fact that glass fiber has more thermal resistance than sisal fiber. The TGA revealed that the sisal or glass fiber composites generated significantly more charred residue than any of the other composites developed [55]. There was some variation in thermal characteristics between sisal with glass hybrid composites made using an extrusion process and those made using an injection molding technique [56]. The research findings point to the importance of fiber dispersion in the matrix for improving the thermal resistance of composites. The influence of particle reinforcement on the thermal characteristics of sisal polyester composite was reported by Venkatram et al. (2016). Comparison research was conducted after adding 3 wt% nanoclay to an existing sisal polyester composite.

The composite without nanoclays was less stable at high temperatures than those reinforced with nanoclay. At 290 °C, the sisal composites lost 82 % of their weight whereas the hybrid composite lost only 79 % of its weight [57]. Charcoal units added to sisal-based composites exerted a positive influence on the deterioration and temperature resistance of the hybrid composite [58]. High degradation temperatures are required to break down the charcoal particles. For this reason, the hybrid mixture with 8, 6, in addition, 4 wt% charcoal retained 30%–40 % of weight regardless of its decomposition at 450 °C. After being heated to 750 °C, the mixture of composite with 8 wt% charcoal retained nearly 38 % of the composite remnant. The thermal characteristics of sisal with SiC-blended composites were reported to be negatively impacted by Teja et al. (2016). The thermal degradation of composites made with 30 wt% sisal fiber and either 5 or 10 wt% SiC particles increased, but the composites still exhibited high thermal stability compared to the pure 30 wt% sisal composites [59]. Sisal, banana plus coir fiber were used to reinforce unique hybrid composites developed by Sumesh et al. (2019). Nanoparticle incorporation improved the thermal resistance of the hybrid composites. Particle reinforcement increased the deterioration temperature of these hybrid composites from 50 % to 70 %. Though particle reinforcement was enhanced by the incorporation of 5 % nanoparticles, mechanical and thermal properties were degraded as an outcome of a greater aggregation of particles [60]. Pappu et al. [61] found that a hybrid composite of polylactic acid (PLA) and sisal and hemp fibers had the same thermal characteristics as pure PLA material. The TGA revealed no change in the thermal properties of the composite after natural fiber was added to the PLA matrix. The hybrid material lost weight in two distinct phases. At temperatures of 300 °C, weight loss began and at 410 °C, it accelerated significantly. At temperatures above 15 °C, the composite began to degrade rapidly and completely, as revealed by Samal et al. [62]. In 2009, Jarukumjorn and Suppakarn explored the rise in heat distortion temperature (HDT) of sisal with glass hybrid PP composites as a function of fiber weight percentage. Adding more reinforcement in the form of sisal fibers raised the HDT. The stiffness of these composites was improved by the addition of the sisal fiber reinforcement, which might account for the observed increases in HDT. The thermal characteristics of a composite made of 10 % sisal fiber with 20 % glass fiber was found to be superior [63].

Govindan and Srinivasan [64] observed identical findings from their study of sisal with basalt hybrid PLA composite. At 250 °C, hybrid composites made of PP and sisal fiber degraded at a lower rate than composites made of sisal fiber alone. Nimanpure et al. [65] reported on the thermal resistance of kenaf with sisal hybrid composites. Thermal degradation was different between composites of different weight percentages. High thermal stability was observed in the hybrid composite made up of 20 % sisal fiber and 20 % kenaf fiber. At 300 °C, this composite lost 10 % of its weight. By improving the fiber–matrix interface, chemical processing of fiber enhanced the thermal stability of the composites. Meenakshi and Krishnamoorthy [66] found that the deterioration temperature of epoxy composites reinforced with sisal, flax, and glass fiber layers was between 306 and 315 °C. Desai et al. [67] showed that carbon epoxy composites benefit from reinforcement with silane-treated sisal fiber. Further, it was reported that the enhanced bonding prevents the free radicals formed during degradation initiation from further spreading. Sisal fiber's degradation temperature is changed when it is reinforced in a hybrid composite because of the removal of organic material during treatment [68]. Maleic anhydride-grafted polypropylene (MAPP) was found to improve the thermal stability of thermoplastic hybrid composites [69]. MAPP was shown to improve the thermal stability of a hybrid polypropylene composite material with coir fiber and sisal fiber combined yarn reinforcement [70]. Asaithambi et al. [71] investigated the thermal characteristics of a banana with sisal fiber–reinforced PLA hybrid composite that had been chemically treated with benzoyl peroxide. The composites' first deterioration along with the final deterioration temperature were slowed by the chemically treated fiber reinforcement.

### 2.2 Jute fiber composites

**Fig. 1:**
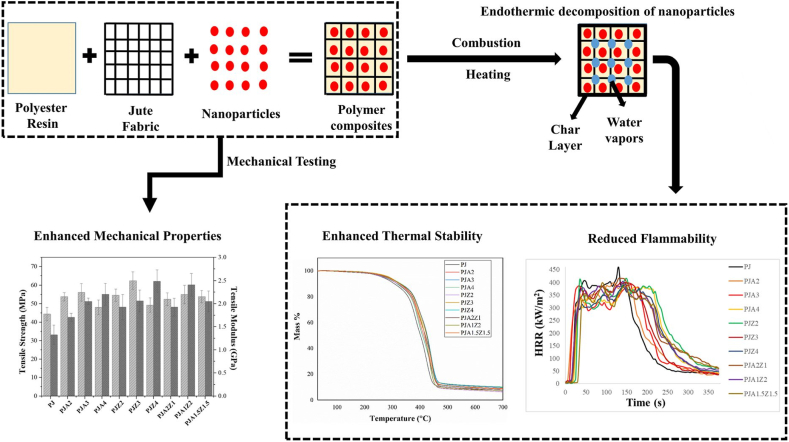
Hydroxide-based nanoparticles addition in jute/polyester composites [72].

Jute fibers are the common reinforcements in polymer matrices and are compatible with almost all other natural fibers and fillers when it comes to hybridization. Composites maintain the 40 % jute fabric weight incorporating the 3 wt% of into a polyester with jute composite significantly improve their tensile, flexural, and impact properties, as shown in Fig. 1. Fourier-transform infrared spectroscopy (FTIR) spectroscopy confirmed the presence of OH connection among ATH, ZHO, with jute fabric, which are responsible for the enhanced mechanical characteristics. After performing a scanning electron microscopy (SEM) examination of the broken surfaces, it was determined that the synergy of the nanoparticles led to enhanced interfacial bonding. The TGA additionally demonstrated that the thermal resistance of the composites improved. Endothermic disintegration regarding ATH and ZHO fine particles as a char layer along with water molecules over combustion significantly improved the flame retardancy for the prepared composites, as evidenced by cone calorimetry along with horizontal igniting tests. By working together, ATH and ZHO particles improve mechanical, thermal, and fireproof qualities of polyester with jute composites [72].

The environmentally beneficial natural fiber–reinforced polylactide composite (NFPC) has been the subject of some attempts to fortify it, but conventional methods reduce its ductility. To simultaneously increase the NFPC's strength and toughness, the authors of the present research synthesized rigid–soft core-shell nanoparticles. Nano-silica along with poly (butyl acrylate) rubber was chosen for their molecular properties, which made them ideal candidates for the core and shell roles, respectively. Crystalline, thermal, as well as mechanical characteristics of NFPC were studied in the presence of core-shell nanoparticles. The findings demonstrated that the core-shell nano-fillers can aid in the formation of a deeper crystalline grain within the PLA matrix and an increase in the material's thermal stability. A bonus is that the elongation at the break of NFPC was not compromised while the nanoparticle's rigid–soft core-shell construction increased the material's strength and stiffness. Furthermore, the resilience enhancement techniques and synergistic effect of core-shell nanoparticles have been demonstrated through field-emission SEM. Toughness is enhanced due to the core-shell filler inducing micro-cracks, shear bands, and matrix fibrillation [73].

### 2.3 Flax fiber composites

Natural fibers are increasingly used in the manufacture of polymer composites, even though these substances have become highly combustible. In this research, titanium dioxide, polydopamine, and diammonium phosphate were added to flax fiber composites made with a polyurethane-based shape memory polymer matrix (PUSMPM) to increase their fire resistance. Polydopamine, a shape memory polymer matrix reinforced using titanium dioxide, was applied to flax fibers that had been processed using diammonium phosphate. The defensive char layer of polydopamine can be seen in SEM images within the generated composites both before and following combustion. Owing to this coating, the composite material burned with much less of a loss of mass than it would have without it. PDA-coated TiO_2_-doped flax fiber composite showed substantial enhancement over uncoated flax fiber composite, with a limiting oxygen index value of 24 % compared to the noncoated flax fiber composite's 20 %. The flame propagation rate was reduced by 81 down to 21 mm/min during the horizontal burning experiment. Using TGA, along with differential thermal analysis, we looked at how polydopamine and titanium dioxide affected fibers and the PUSMPM. Titanium dioxide, polydopamine, and diammonium phosphate were shown in this investigation to significantly increase the combustibility of natural fiber composites made from a PUSMPM [74].

### 2.4 Poplar fiber composites

Impact of nanoscale iron oxide (Fe_2_O_3_) particles on medium-density fiberboard (MDF) physical and chemical characteristics was studied. In this investigation, experimented with untreated poplar fibers along with a variety of nano iron oxide loadings (0.5, 1.5, and 2.5 wt%). Before being embedded into natural fibers, Fe_2_O_3_ nanoparticles were mixed into urea formaldehyde resin using a high-vacuum mechanical stirrer. Dry mat layers were created by winding untreated poplar fibers onto metal frames. Twenty distinct composite specimens were created. Thickness swelling, retention of water, moisture content, and density were measured for each composite specimen using industry standards EN-322, EN-317, EN-323, and ASTM D570. Results showed that using iron oxide nanoparticles that were uniformly dispersed greatly enhanced thickness swelling (*T*_s_). At the highest loading, 3 wt%, water absorption (WA) increased by as much as 49.2 % and 34.5 %, respectively. This study used SEM to examine the nanoparticles' microstructure and determine whether or not iron oxide nanoparticles demonstrate favorable interactions with urea formaldehyde along with poplar wood fibers. A differential scanning calorimetry (DSC) and a TGA were performed to look into the effects of Fe_2_O_3_ nanoparticles on heat and mass transfer. Fe_2_O_3_ improved the resin's curing temperature as well as thermal stability. To regulate the dissemination of Fe_2_O_3_ a one-way analysis of variance was created. As a result, the incorporation of iron oxide nanoparticles into an epoxy polymer results in a more rigid matrix, which in turn increases nano-MDF's potential for exhibiting improved physical characteristics [75]. Table 1 explains the types of nanoparticles reinforced with natural fiber-based composites.

**Table 1:** Types of nanoparticles in natural fiber–reinforced composites.

Nanoparticle	Concentration (wt.%)	Natural fiber Reinforcement	Matrix	Ref.
Nanoclay	0, 1, 3, 5	Sisal	Polypropylene	[76]
	0, 2, 4	Bagasse	High-density polyethylene (HDPE)	[77]
	1–4	Bagasse	Polypropylene	[78]
	0, 1, 3, 5	Sisal	Epoxy	[79]
	0, 2, 4	Bagasse	Polyethylene	[80]
	0, 1, 3, 5	Bamboo	High-density polyethylene (HDPE) + malleated polyethylene (MAPE)	[81]
Bacterial cellulose	Surface treated	Sisal	Acrylated epoxidized soybean oil (AESO)	[82]
		Hemp	Poly (l-lactic acid) + cellulose acetate butyrate (CAB)	[83]
		Sisal	Acrylated epoxidized soybean oil (AESO)	[84]
MWCNTs	0.1, 0.5, 1	Palm oil	Epoxy	[85]
Oil palm CNTs	0, 0.5, 1-2	Flax	Epoxy	[86]
Graphene	0.01–0.05	Palm oil	Epoxy	[87]
Nanoclay	0, 5, 8, 10	Flax	Epoxidized soybean oil (ESO)	[88]
Titanium dioxide, TiO_2_	0–4	Jute	Epoxy	[89]
Zinc oxide, ZnO	0–4	Jute	Epoxy	[90]
Silica, SiO_2_	0, 1.5, 3	Flax	Polypropylene (PP)	[91]
	0, 5	Sisal	Polyester	[92]
Zirconium oxide, ZrO_2_	Grafted	Flax	Poly (lactic acid)	[93]
Graphene	–	Bagasse	Polypropylene	[94]
	0–5	Kenaf		[95]
Alumina, Al_2_O_3_	0–3	Flax/PLA	Epoxy	[96]
MWCNTs	0–0.6	Ramie	Epoxy	[97]
Titanium dioxide, TiO_2_	0–7	Flax	Epoxy	[98]
Oil palm CNTs	0.15	Bamboo	Epoxy	[99]
Silica, SiO_2_	0, 2, 5	Bagasse	High-density polyethylene (HDPE)	[100]
Nanoclay	0–1.5	Hemp	Unsaturated polyester (UP) + epoxidized methyl soyate (EMS)	[101]
Titanium dioxide, TiO_2_	0, 2	Bagasse	Ethylene co-vinyl acetate (EVA)	[102]
Nanoclay	1, 2.5, 5, 10	Hemp	Poly (lactic acid)	[103]
Cenosphere	–	Bamboo	Epoxy	[104]
Bacterial cellulose	Surface treated	Sisal	Poly (l-lactic acid) + cellulose acetate butyrate (CAB)	[105]
Oil palm CNTs	–	Kenaf	Epoxy	[106]
	0, 0.5, 1–4	Hemp		[107]
Graphene	0.3, 1, 3	Jute	Epoxy	[108]
Alumina, Al_2_O_3_	0, 5, 10, 15 (μm)	Jute	Epoxy	[109]
	10 (μm)	Coir		[110]
Titanium dioxide, TiO_2_	Grafted	Flax	Poly (lactic acid)	[111]
Exfoliated graphite, xGnp	0, 1, 3, 5	Kenaf	Poly (lactic acid)	[112]

## 3 Thermal characteristics of nanoparticle-based hybrid composites

Graphite is composed of numerous layers of carbon atoms piled on top of each other. Graphene is a monolayer of graphite that can be mechanically exfoliated. The material exhibits notable characteristics such as exceptional strength, low mass, and excellent resistance to corrosion. In comparison to metals, it also has superior electrical and thermal conductivities. Additionally, its remarkable sensitivity renders it suitable for use in nanosensor technology. The advantageous characteristics of graphene, such as its lack of metallic contaminants and two-dimensional structure, make it a valuable material in the field of nanofabrication. Graphene can be synthesized using various techniques, such as mechanical exfoliation, intercalation, chemical synthesis, and burning of agricultural waste materials. This substance finds utility in a diverse range of applications. Graphene exhibits superior thermal conductivity compared to copper, enabling more effective heat dissipation from miniature electronic devices such as computer chips. Numerous industries anticipate the use of materials or nanocomposites that possess distinctive features. The integration of graphene into polymers has been found to considerably enhance the attributes of composites, including electrical conductivity, mechanical strength, thermal stability, and fire resistance [113]. It effectively prevents the initiation and propagation of cracks when subjected to periodic loading. The results of the fatigue studies indicate that after 800 cycles, the hybrid composite material consisting of bamboo, glass fibers, and nanoparticles exhibits a load capacity that is 22.5 % greater than that of all other composite mixtures. The fatigue life of hybrid composites exhibits an upward trend when the filler dimension decreases.

**Fig. 2:**
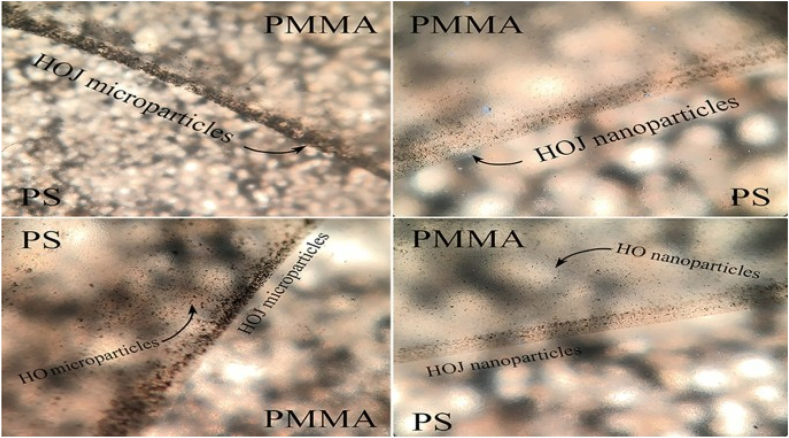
OM images depicting the presence of HOJ nanoparticles in PMMA/PS polymer blends [115].

The thermal resistance of the hybrid bamboo/glass FRP composite with nanoparticles was enhanced, as indicated by the findings of TGA/DTG studies [114]. The investigation examines the features of the micro- and nanoparticles of hydrophobic graphene oxide (HO) and HO Janus (HOJ) by the use of several analytical techniques, including field-emission scanning electron microscopy, TGA, energy dispersive X-ray spectroscopy, Fourier transform infrared spectroscopy, and contact and centrifuge angle tests. Various micro- and nanocomposite samples of PS/PMMA were fabricated using solution mixing techniques and subsequently evaluated through thermal conduction experiments. Fig. 2 depicts the optical microscopic (OM) images of the PMMA/PS blends containing HOJ nanoparticles. The findings of the study indicate that the incorporation of HO micro- and nanoparticles in either phase of the PS/PMMA blend resulted in a slight increase in the conduction coefficient (K). However, it was observed that the combined presence of these particles in both phases had a more substantial effect on K. In contrast, while evaluating the outcomes of the blend specimens with and without the incorporation of HOJ micro- and nanoparticle, a significant enhancement in the heat conductivity of the polymer with polymer interface was seen as a result of the inclusion of Janus particles. Furthermore, it was observed that the thermal resistance of the samples exhibited a reduction as their thermal conductivity increased. This trend was particularly pronounced in the samples that contained both HO and HOJ micro- and nanoparticles [115].

## 4 Thermal behavior of nanocomposites

Nanoparticle-based polymer composites are potential candidates for various engineering applications apart from natural-fiber and hybrid composites. Polymer composites are normally reinforced with organic or inorganic nanoparticles and were assessed for their physical and thermal properties in most cases. Cellulose-based nanocomposites and carbon or graphene-based nanocomposites were analyzed by numerous researchers for their thermal and electrical properties [[116], [117], [118], [119]]. Bioactive polymeric nanocomposites are materials that are considered essential and have gained significant interest due to their wide range of applications within the human organism. A complex of poly (hydroxyethyl methacrylate) (pHEMA) was fabricated using hydroxyethyl methacrylate (HEMA) as the primary material with the aid of a twin-screw extruder. The nanocomposites based on pHEMA were fabricated by incorporating titanium oxide nanoparticles (1 wt % TiO_2_) and graphene oxide (0.1 wt% GO) and their thermomechanical properties were investigated. The PT and PTG nanocomposites exhibited notable alterations in microstructural behavior, resulting in enhanced thermomechanical characteristics as compared to pure pHEMA. This study investigated the suitability of pure pHEMA as well as PT and PTG nanocomposites for implantation in dental implications [120].

A few experimental works used betel nut husk fiber (BNHF) derived nanocellulose reinforced in polyvinyl alcohol (PVA) matrix with various weight fractions and their thermal behaviour was evaluated using TGA and DSC techniques. It was understood from the results that the addition of nanocellulose to PVA matrix enhanced thermal stability of the nanocomposites due to the establishment of strong hydrogen bonding between the composite constituents [121]. In some other studies, vinyl laurate micro fibrillated cellulose (VL-MFC) were reinforced in PLA matrix and their rheological and thermal properties were evaluated. It was observed from the results that the thermal stability of the PLA matrix was increased due to the addition of VL-MFC while the storage and loss modulus of the VL-MFC nanocomposites increased with the addition of the micro cellulose. These changes were supported by the better interfacial adhesion between the nanoparticles and the matrix and due to the liquid-to-solid transition in the composites when subjected to change in temperatures [122]. From the above discussions, it could be understood that the thermal stability of the polymer composites were improved by the incorporation off the nanoparticles.

The composite polymer electrolytes are specifically designed to be compatible with the 3D printing process known as fused deposition modelling. The composite materials used in this study were composed of altered poly (ethylene glycol) (PEG), lithium bis-(trifluoromethylsulfonyl) imide (LiTFSI), and nanofillers based on SiO_2_. The end group modification of PEG was effectively carried out, resulting in the production of telechelic PEG that incorporates either ureidopyrimidone (UPy) or barbiturate moieties. These modified PEG molecules possess the ability to form supramolecular networks through hydrogen bonding interactions, thereby providing self-healing properties to the electrolyte system. Silica NPs were used as a filler to modify the mechanical properties of the electrolyte to facilitate its 3D-printing capability. It is anticipated that the surface modification of the NPs using an ionic liquid (IL) or hydrophobic alkyl chains will result in an enhanced dispersion of the NPs within the polymer matrix. The rheological properties of composites containing varying concentrations of nanoparticles (in increment of 5 %, from 5 % to 15 %) and LiTFSI salt were examined to acquire an in-depth understanding of their suitability for 3D printing. Additionally, the ionic conductivity of these composites was assessed using broadband dielectric spectroscopy. The successful 3D printing of the composite electrolyte, PEG with other constituents in combination with 15 % NP-IL, demonstrates its potential as a viable option for printable composite electrolytes. TGA experiment was performed using the Netzsch TG 209 F3 instrument. Samples weighing between 5 and 10 mg were carefully positioned into alumina crucibles. These crucibles were then subjected to controlled heating in an environment free of reactive gases, with a heating rate of 10 K^−1^.

**Fig. 3:**
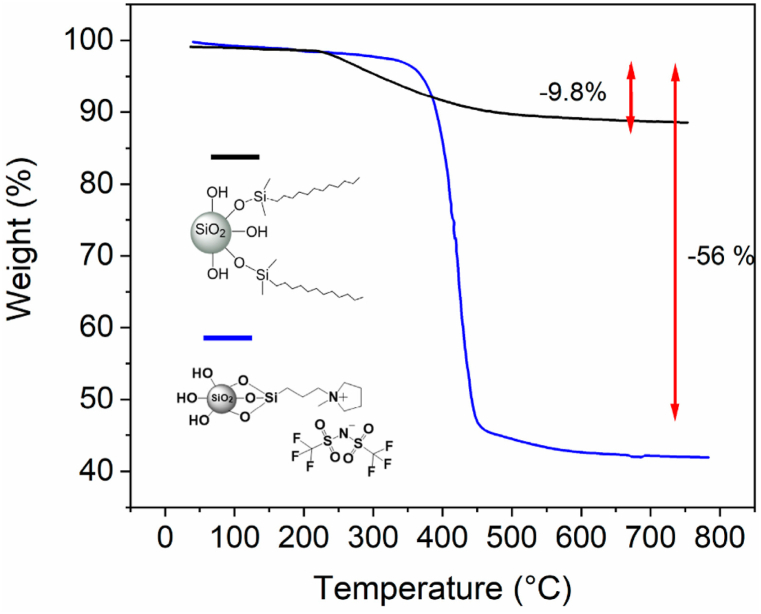
Thermal degradation curves of nanocomposites with silica nanocomposites with surface modification done using ionic liquids (blue line) and Alkyl compounds (black line) [124].(For interpretation of the references to colour in this figure legend, the reader is referred to the web version of this article.)

DSC measurements were conducted using a Netzsch DSC 204 F1 instrument. Before measurement, the samples were subjected to an air-drying process in a vacuum environment at 80 °C. Subsequently, the dried samples were carefully positioned in aluminum pans and measurements were carried out in the presence of a nitrogen atmosphere. The thermal history of the samples was eliminated with the application of heat at 100 °C. The cooling process was conducted until a temperature of −20 °C was reached, with a cooling rate. Subsequently, heating curves were recorded up to a temperature of 170 °C, using a heating rate. The samples underwent a drying process at 80 °C under vacuum conditions for 24 h. Subsequently, measurements of shear rate vs viscosity were conducted at various temperatures. The silica nanopowder, which incorporated alkyl groups (referred to as NP-alk), demonstrated the stretching vibration of the alkene of C–H within the range of approximately 3000 cm^−1^. The findings from the thermogravimetric study are illustrated in Fig. 3. The nanoparticles that underwent modification exhibited a reduction in weight when exposed to elevated temperatures, which can be attributed to the breakdown of the organic modifiers present on their surfaces. This outcome serves as evidence of the effective functionalization of the nanoparticles. The Ludox SM30 nanoparticles functionalized with ionic liquid groups (referred to as NP-IL) exhibited thermal stability up to 350 °C, consistent with previous findings reported in the literature for nanoparticles functionalized with ILs [123]. In contrast, the thermal stability of NP-alk was found to be considerably lower, reaching only up to 250 °C. The estimation of the size of the nanoparticles (with a range of 10–120 nm) was conducted using dynamic light scattering and TEM measurements. Furthermore, proton nuclear magnetic resonance (^1^H NMR) and solid-state cross-polarization/magic angle spinning silicon-29 nuclear magnetic resonance (CP/MAS 29Si-NMR) techniques were also used to characterize NPs [124].

## 6 Summary and conclusions

Natural fiber composites have become the first-choice materials in various engineering applications due to their economic feasibility, technicality and environmental friendliness. Though many research works were undertaken for developing novel composite materials using natural fibers, various technical and economic constraints retarded the full-scale implementation of natural fiber composites in different commercial applications. A high level of priority must be given to the thermal ability of the natural fiber composites during their processing and the retention of their inherent properties even after exposure to thermal and other heat-related environments. In this context, the thermal analysis of the composite materials paved way for the development of newer and novel composite materials if the selection process of the materials is rightly optimized. Various approaches were analyzed in the previously published literature to improve the thermal properties of the natural fiber–reinforced polymer composites. Some of the commonly adopted techniques were tailoring the fiber and matrix type, addition of organic and inorganic fillers specifically in nanoscale, orientation, chemical modification, and loading of the natural fibers. In most of the studies, it was pointed out that the addition of nanoparticles resulted in a better enhancement of the thermal properties of the composites.

Some of the commonly analyzed thermal behavior includes the mass loss of the nanoparticles reinforced composites when heated, the temperature of complete degradation of the composites, their glass transition temperature, and the viscoelastic behavior of the composites. The addition of nanoparticles facilitated the composites to attain better thermal properties. Specifically, the viscoelastic properties of the natural fiber–reinforced polymer composites could be readily improved by the addition of nanoparticles, which promote good interfacial adhesion between the matrix and the reinforcements and improve the energy dispersion. The addition of nanoparticles also governed the thermal degradation of the polymer composites by enhancing the molecular mass of the composites, increasing the cross-linkages, and increasing tacticity, which in turn affects the glass transition properties of the composites. Additionally, the hybridization of the natural fiber composites with the nanoparticles improved the crystallization kinetics, heat of fusion, heat capacity, melting behavior, and liquid crystal transition temperature, which enhance the thermal characteristics of the composites. From all the above studies, it could be concluded that the nanoparticles incorporated in natural fiber composites exhibit better thermal behavior and are highly suitable as renewable materials for various high-temperature applications.

## CRediT authorship contribution statement

**D. Balaji:** Writing – original draft, Supervision, Data curation, Conceptualization. **P. Sathish Kumar:** Writing – review & editing, Writing – original draft, Supervision, Data curation, Conceptualization. **V. Bhuvaneshwari:** Writing – original draft, Supervision, Data curation, Conceptualization. **L. Rajeshkumar:** Writing – original draft, Supervision, Data curation, Conceptualization. **Manoj Kumar Singh:** Writing – review & editing, Supervision, Data curation, Conceptualization. **M.R. Sanjay:** Writing – review & editing, Supervision, Funding acquisition, Conceptualization. **Suchart Siengchin:** Writing – review & editing, Supervision, Conceptualization.

## Consent to participate

Not applicable.

## 5 Future scope

The trend of using nanoparticles for enhancing the thermal properties of the natural fiber–reinforced polymer nanocomposites was analyzed using a patent database due to its frequently updated nature. This analysis provides a path for researchers in this domain to take direction on decision making of their research work. This also paves a pathway to critically assess the application point-of-view of the material through the identification of the innovations using this material [125,126]. It could be understood from many of the earlier research works that natural fiber composites with nanoparticles as hybrid reinforcements possess better thermal behaviour and thermal stability for various engineering applications. Since the thermal behaviour of the nanoparticle-reinforced natural fiber composites were found to be better, they can capture a significant market share in electrical and sports applications. To further enhance the potential of the nanoparticle-reinforced natural fiber composites, the weathering behaviour of the composites specifically in outdoor environments must be assessed in detail. Exposure of the composites to sunlight, moisture and UV light can be monitored and their behaviour can be evaluated under such conditions to enhance the applications spectrum of the composites [127,128].

Table 2 reveals impact of natural fiber with nanoparticles searched in English all categories, with keywords “natural fiber reinforced composites” and “nanoparticles”. Eight keywords were searched. The researchers in this domain can focus on the International Patent Classification (IPC) categories C04B, B29C, C08J, and C08L. In Over the past decade, there has been slow but steady progress. The recent inclusion of advanced materials suggests the potential for novel nanoparticles to introduce newer application. Furthermore, only two countries have contributed to this technology, indicating substantial opportunities for growth and development in the future [129].

**Table 2:** Patent landscape analysis [129].

S. No.	Countries	Count	IPC	Count	Year	Count
1	United States of America	3	C04B	3	2014	1
2	Patent Cooperation Treaty	3	B29C	2	2015	1
3	India	2	C08J	2	2016	0
4			C08L	2	2017	1
5			A61F	1	2018	0
6			A61L	1	2019	0
7			B01D	1	2020	1
8			B01J	1	2021	1
9			B28B	1	2022	1
10			B29D	1	2023	0

## Availability of data and materials

Not applicable.

## Ethics approval

Not applicable.

## Consent for publication

This manuscript does not contain any individual person's data in any form.

## Funding

Not applicable.

## Declaration of competing interest

The authors declare the following financial interests/personal relationships which may be considered as potential competing interests:The author(s) of this paper, L. Rajeshkumar and M. R. Sanjay, works as an Associate Editor at Heliyon Materials Science. If there are other authors, they declare that they have no known competing financial interests or personal relationships that could have appeared to influence the work reported in this paper.
